# Ocular social jetlag: a driver of immune-metabolic dysfunction in dry eye disease

**DOI:** 10.3389/fimmu.2026.1771774

**Published:** 2026-02-20

**Authors:** Yating Zhou, Yuliang Gu, Jian Yin, Tuo Jin

**Affiliations:** Department of Ophthalmology, Kunshan Hospital of Traditional Chinese Medicine, Kunshan, Jiangsu, China

**Keywords:** circadian rhythm, dry eye disease, inflammation, ocular surface, social jetlag

## Abstract

Dry eye disease (DED) is increasingly prevalent among young individuals and often exhibits severe symptoms despite minimal structural damage, challenging the traditional structure–inflammation paradigm. We propose the concept of ocular “social jetlag,” defined as chronic circadian misalignment imposed by modern lifestyles, as a key upstream driver of meibomian gland dysfunction and contemporary DED. We integrate emerging evidence to suggest that social jetlag disrupts peripheral ocular clocks, triggering immune–metabolic circadian reprogramming characterized by metabolic stress, loss of temporal immune gating, oxidative amplification, and inflammasome activation. This cascade precedes overt tissue damage and explains the mismatch between symptoms and structural findings. Viewing the ocular surface as a dynamic biosensor of systemic clock–immune–metabolism networks, we further highlight digital immune phenotyping and chronotherapeutic interventions as promising strategies for precision management. This framework reframes DED from a purely local disorder to a rhythm-driven systemic condition, opening new avenues for mechanism-based prevention and treatment.

## Highlights

- Proposes “ocular social jetlag” as a key upstream driver of contemporary dry eye disease.
- Describes a cascade of immune-metabolic circadian reprogramming triggered by clock disruption.
- Advocates for a paradigm shift from local treatment to rhythm-focused precision management.

## 1 Introduction: the temporal dimension of ocular homeostasis

Dry eye disease (DED) has traditionally been regarded as a degenerative condition that worsens with age, primarily driven by insufficient tear production or excessive evaporation. However, recent epidemiological data reveal a rapid increase in DED among younger populations, demonstrating strong associations with modern lifestyle factors: sedentary behavior, intense screen use, high-fat diets, and irregular sleep patterns are all closely linked to its onset (1, 2). Notably, many young patients present with severe symptoms and exhibit limited response to conventional treatments, despite showing no significant structural damage to the meibomian glands (3–5). These observations suggest that the traditional “structure-inflammation” model is insufficient to explain this emerging, lifestyle-driven DED phenotype.

In addition to the lifestyle-related factors discussed above, mechanisms such as neuropathic pain sensitization (which may drive central sensitization and hyperalgesia) (6), psycho-somatic influences (e.g., stress and anxiety that disrupt neuroendocrine homeostasis) (7), sex hormone regulation (particularly evident in female patients) (8), and environmental exposures (e.g., air pollutants that directly provoke inflammation) (9) have also been investigated in dry eye disease pathogenesis. These perspectives offer important insights into explaining symptom-sign discordance and chronic disease progression. However, most existing perspectives consider these factors as relatively independent pathogenic units, making it difficult to systematically explain their time-dependent fluctuations under modern lifestyle conditions or the potential coupling and synergy among distinct pathological processes.

In this context, chronobiology offers a critical explanatory framework for this clinical challenge. Chronobiology offers a crucial explanatory framework for this clinical conundrum. Accumulating evidence indicates that ocular surface homeostasis is highly dependent on circadian regulation: lipid secretion from meibomian glands, metabolic activity of the lacrimal gland, and immune surveillance at the ocular surface all exhibit strict temporal characteristics. When these timing mechanisms are disrupted, functional imbalance can drive disease onset even in the absence of structural damage (10, 11).

Modern lifestyles commonly impose chronic circadian stress, including exposure to artificial light at night, shift work, unstable sleep patterns, and weekday-weekend sleep discrepancies, collectively termed “social jetlag (SJL).” Various chronic conditions such as metabolic syndrome, depression, and chronic pain are closely associated with this implicit rhythm disturbance (12–14). In recent years, the link between circadian disruption and ocular surface diseases has garnered increasing attention. The eye plays a unique dual role in the circadian system: on one hand, it serves as the primary pathway for light signal input, regulating the master clock (suprachiasmatic nucleus, SCN) via intrinsically photosensitive retinal ganglion cells (ipRGCs); on the other hand, the cornea, conjunctiva, lacrimal gland, and meibomian glands all possess autonomous molecular oscillators, independently regulating secretion, barrier maintenance, and immune surveillance (15, 16). This makes the eye both a source of circadian synchronization and highly susceptible to the direct impact of circadian misalignment.

We therefore propose that social jetlag may disrupt ocular peripheral clocks, inducing “immune-metabolic circadian reprogramming,” thereby constituting a crucial upstream mechanism for contemporary DED and meibomian gland dysfunction. This hypothesis reintegrates the circadian-immune-metabolic axis, providing a novel framework for understanding and intervening in modern ocular surface diseases.

## 2 Social jetlag: a primary environmental driver of chronic circadian stress at the ocular surface

SJL refers to a chronic, systematic misalignment between an individual’s endogenous circadian clock and societally imposed schedules. More than mere sleep deprivation, it represents a state of chronic circadian desynchrony driven by conflicting environmental cues such as light exposure, food intake, and activity patterns (17). For the ocular surface, a tissue that directly receives light input and harbors robust peripheral clocks, SJL acts as a persistent stressor that disrupts the precise temporal order essential for maintaining immune homeostasis.

Quantified as the difference in sleep midpoint between workdays and free days, SJL has been established as an independent, dose-dependent risk factor for both DED and MGD. For instance, a large-scale study of Chinese adolescents reported a 28% increase in DED risk for every additional hour of SJL (18). Individuals with significant SJL are typically evening chronotypes, which forces the ocular surface to sustain high functional loads during its biological “rest and repair” phase. During this time, parasympathetic-driven basal tear secretion is minimal, and attentional blink suppression further reduces lubrication, creating a severe evaporation-secretion imbalance. This physiological mechanism explains the intense ocular dryness following prolonged evening screen use and the characteristic morning irritation (19, 20).

Furthermore, nocturnal exposure to blue light (405–480 nm) presents a dual threat. Beyond suppressing melatonin via the ipRGC-SCN axis, it directly penetrates the cornea to impair circadian gene expression in epithelial and meibomian gland cells. Prolonged exposure (2–3 hours) can trigger mitochondrial ROS production and NLRP3 inflammasome assembly, driving direct epithelial damage and inflammation (21–23).

The meibomian gland clock is highly sensitive to feeding signals. High SJL, often associated with late dinners (>21:00) and erratic high-fat intake, disrupts this metabolic entrainment. This mistiming abnormally activates lipid synthesis genes (e.g., Fasn, Scd1), shifting meibum composition toward saturated fatty acids with a higher melting point, a key metabolic precursor to duct obstruction (24). In summary, social jetlag is a multidimensional circadian stressor. By disrupting the phase and amplitude of ocular surface clocks within weeks, it opens a critical window for the immune-metabolic reprogramming that drives disease.

## 3 The circadian basis of ocular surface homeostasis: temporal programming of immune function

Ocular surface homeostasis depends on a multi-tissue temporal program orchestrated by the endogenous circadian clock. This program governs not only diurnal variations in secretion and barrier function but also imposes strict temporal control over the surveillance, defense, and tolerance states of innate and adaptive immunity.

The cellular rhythms of all ocular surface tissues originate from cell-autonomous molecular clocks. A core transcription-translation feedback loop drives these rhythms, in which the CLOCK: BMAL1 heterodimer activates genes like Period and Cryptochrome, whose protein products later feedback to suppress their own transcription, generating 24-hour oscillations (25–27). Nuclear receptors REV-ERBα/β and ROR fine-tune this loop by regulating Bmal1 expression, thereby stabilizing the rhythm and serving as a key interface linking the core clock to downstream metabolic and immune pathways (28–30).

Under normal conditions, a coordinated temporal division of labor exists across ocular surface tissues. The lacrimal gland peaks its basal secretion during the active phase and upregulates immune effectors like lysozyme several hours before waking, establishing a state of predictive immune readiness for daytime challenges (11, 31–34). At night, TLR signaling and NF - κB activity are attenuated to promote a reparative state.

In the meibomian glands, key lipid synthesis enzymes, regulated by the BMAL1–REV−ERB axis, peak in the early active phase to produce high - quality meibum that stabilizes the tear film (35, 36). Circadian disruption alters this lipid profile, leading to peroxidation products that trigger low-grade inflammation (37).

The cornea and conjunctiva exhibit time-gated barrier functions: epithelial turnover and mucin expression are rhythmically controlled to maximize daytime defense, while resident immune cells modulate their activity and metabolism to balance daytime vigilance with nighttime repair and tolerance (38–40).

Although each tissue maintains local oscillations, their phases are synchronized daily by the suprachiasmatic nucleus via neural and humoral pathways. Meal timing also serves as a potent zeitgeber, particularly for the meibomian gland clock (41–43). This evolutionarily conserved temporal programming enables the ocular surface to anticipate and efficiently adapt to daily environmental cycles, a form of predictive physiology that minimizes immunopathological cost. Its integrity, however, relies entirely on stable synchronization between internal rhythms and external time cues. Social jetlag undermines this synchronization, disrupting the very temporal foundation of ocular surface immune homeostasis.

## 4 Immunometabolic circadian reprogramming: a cascade from clock misalignment to homeostatic collapse

**Figure 1:**
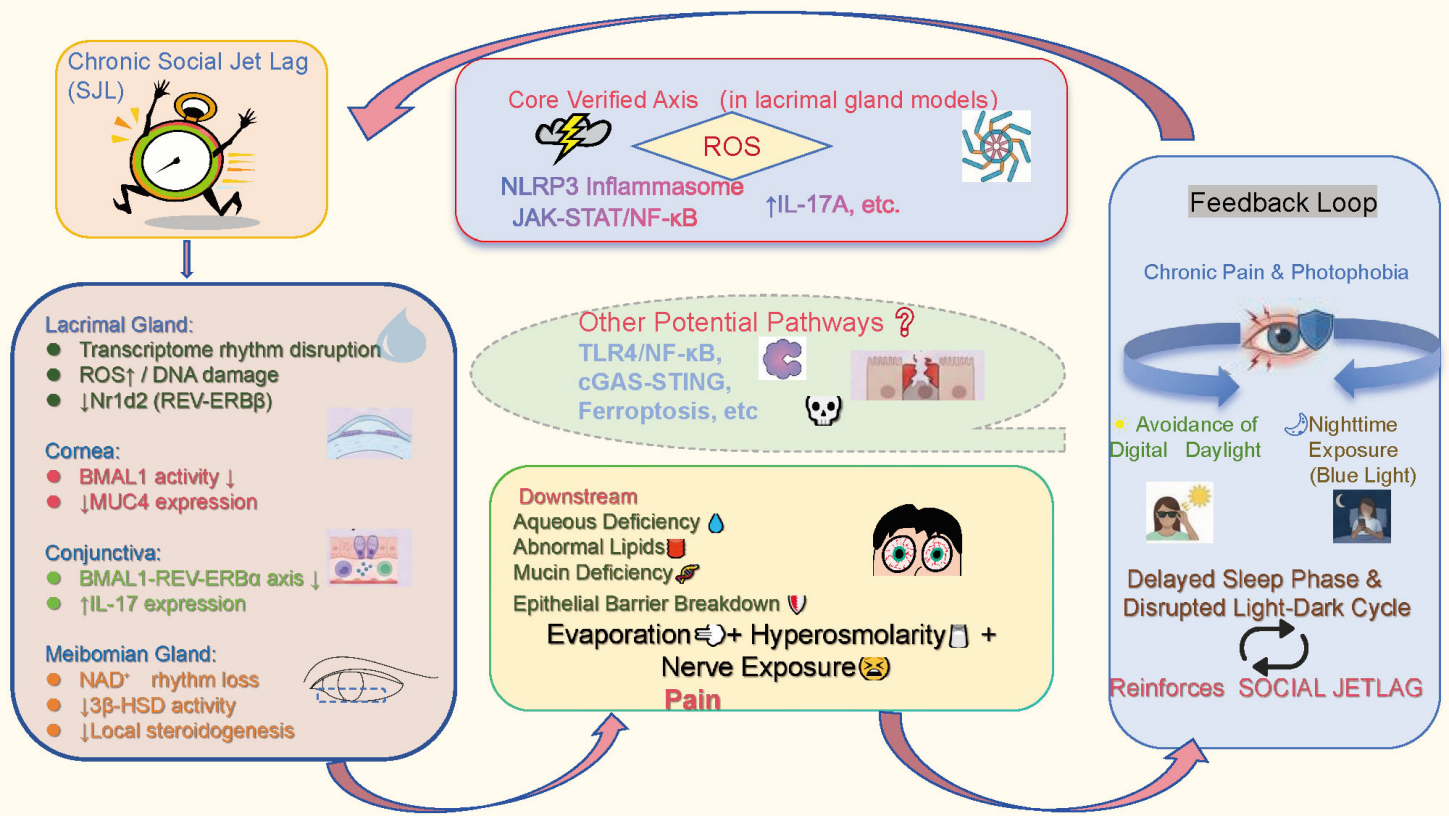
A cascade from multi-tissue clock disruption to ocular surface homeostatic collapse. Figure  outlines a mechanistic model linking chronic SJL to ocular surface homeostatic collapse, comprising upstream clock disruption, midstream inflammatory amplification, downstream decompensation, and a feedback loop. Upstream (left): SJL induces tissue-specific changes, including lacrimal gland transcriptional remodeling, ROS increase, and Nr1d2 downregulation; corneal BMAL1 and MUC4 decrease; conjunctival BMAL1–REV-ERBα axis suppression and IL-17 upregulation; meibomian gland NAD^+^ rhythm loss, 3β-HSD decline, and impaired steroidogenesis. Midstream (central): The core verified axis (red solid box), validated in lacrimal models, is ROS → NLRP3 inflammasome → JAK-STAT/NF-κB → IL-17A. Potential pathways (dashed cloud), including TLR4/NF-κB, cGAS-STING, and ferroptosis, are linked by grey dashed arrows, indicating indirect support. Downstream cell death modalities (e.g., pyroptosis/ferroptosis) remain untested in ocular SJL models. Downstream (lower): Convergent functional failure leads to aqueous, lipid, and mucin deficiency; epithelial barrier breakdown; evaporation-driven hyperosmolarity; nerve exposure; and pain. Feedback loop (right): Chronic pain and photophobia reduce daytime activity and increase nighttime blue-light exposure, delaying sleep phase and thereby reinforcing SJL. Line code: Red solid lines denote validated pathways; grey dashed lines indicate hypothetical links.

To clarify the potential causal relationship between SJL and DED, we propose an integrative pathological framework termed immunometabolic circadian reprogramming. This concept describes a process whereby chronic SJL disrupts the intrinsic circadian regulation of immune surveillance and cellular metabolism across ocular surface tissues, progressively shifting their functional set points and ultimately driving tissue‐level homeostatic imbalance. Rather than representing a simple linear pathway, this process comprises dynamic interactions with both feedforward drivers and feedback reinforcement. Based on available evidence, we divide this cascade into three interlinked stages: upstream tissue-specific clock disruption, midstream convergence and amplification of metabolic–immune signaling, and downstream decompensation of ocular surface homeostasis (**Figure 1**). To avoid overinterpretation, experimentally supported mechanisms are distinguished from hypothetical links throughout the text and summarized in **Table 1**.

**Table 1:** Experimental evidence supporting the immunometabolic circadian reprogramming cascade.

Stage & pathway	Key mechanistic finding	Experimental model	Evidence strength
UPSTREAM: clock disruption			
Lacrimal gland rhythm loss	>2000 genes lose rhythmicity; delayed immune cell trafficking.	Chronic phase-advance mice	Direct (SJL model) (44)
Oxidative gland damage	ROS↑, γ-H2AX↑, acinar atrophy & irreversible hyposecretion.	Sleep deprivation mice	Direct (SJL model) and Direct (circadian disruption model) (45)
REV-ERBβ metabolic target	Nr1d2 downregulation impairs metabolism; agonist restores function.	Postoperative dry eye mice	Circadian gene manipulation (DED context) (11, 46)
Corneal BMAL1-MUC4 axis	BMAL1 directly regulates MUC4; loss disrupts barrier.	SJL & Bmal1 KO mice	Direct (39)
Conjunctival BMAL1-REV-ERBα-IL-17 axis	Clock disruption derepresses IL-17; REV-ERBα agonist suppresses it.	SJL & Bmal1-defective mice	Direct (47)
MIDSTREAM: amplification			
Core axis: ROS-NLRP3-IL-17A	ROS → NLRP3 → p-JAK2/STAT3/NF-κB → IL-17A↑, causing damage.	Sleep deprivation + nanoparticle mice	Directly Validated (48)
TLR4/NF-κB pathway	Co-activated with NLRP3 in diabetic dry eye; suppressed by melatonin.	Diabetic dry eye & NASH mice	Indirect (DED/metabolic models) (49, 50)
cGAS-STING pathway	Activated in DED; sleep loss triggers it via mtDNA in other tissues.	DED & sleep-deprived models	Indirect (cross-tissue circadian inference) (51–53)
Ferroptosis hypothesis	Occurs in DED cornea; Bmal1 loss increases susceptibility in TBI.	DED rat & TBI mouse models	Hypothetical (Cross-system evidence) (54, 55)
DOWNSTREAM: failure			
BMAL1-ITPR2/3 secretion axis	BMAL1 directly regulates ITPR2/3; deficiency causes hyposecretion.	Bmal1 KO rats	Direct (56)
Multi-tissue uncoupling (time-window)	Hypoxia remodels clock, causing synchronized barrier/nerve/immune peak damage at ZT18.	Hypoxia mouse model	Direct (57)
Meibomian gland NAD^+^-3β-HSD axis	Circadian NAD^+^ regulates 3β-HSD/local androgens; NMN restores function.	Mouse & human MG models	Mechanistically Defined (58–60)
FEEDBACK: chronicity			
Epigenetic inflammation-to-clock feedback	DED inflammation causes PER2/3 hypomethylation & upregulation.	Experimental DED mice & *in vitro*	Direct (61)

### 4.1 Upstream: SJL-induced multitissue clock dysregulation and functional impairment

SJL perturbs light–sleep–feeding cycles, thereby disturbing local clocks in the lacrimal gland, meibomian gland, cornea, and conjunctiva. Because these tissues differ markedly in function, the initial manifestations of clock disruption are tissue specific.

In the lacrimal gland, which depends strongly on circadian regulation for secretory activity, chronic phase-advance models reveal widespread remodeling of the circadian transcriptome, with more than 2,000 rhythmic transcripts showing phase shifts or loss of oscillation, including genes involved in metabolic and immune pathways. This disturbance is accompanied by attenuation of diurnal variation in gland weight, acinar cell size, and pilocarpine-stimulated tear secretion (44). Prolonged circadian disruption or sleep deprivation further leads to excessive accumulation of ROS, upregulation of the DNA damage marker γ-H2AX, and structural abnormalities of acini, culminating in irreversible tear hyposecretion (45). Mechanistic evidence is provided by postoperative dry eye models, in which surgical stress selectively downregulates Nr1d2 (REV-ERBβ) in the lacrimal gland, impairing mitochondrial function and lipid metabolism; pharmacological activation of REV-ERB reverses these metabolic defects and restores tear secretion (11, 46). Corneal nerve injury models further demonstrate that peripheral neural input is required to maintain lacrimal clock integrity, whereas metabolic disease models (db/db mice) show that systemic metabolic dysfunction alone can abolish approximately half of rhythmic lacrimal genes and advance the phase of the remaining oscillations (62, 63).

Corneal and conjunctival homeostasis relies on precise temporal gating. In the cornea, BMAL1 directly regulates transcription of the transmembrane mucin MUC4, and both chronic circadian misalignment and Bmal1 deficiency markedly reduce MUC4 expression, compromising tear film stability and epithelial barrier integrity; supplementation of MUC4 or restoration of BMAL1 activity by melatonin improves barrier function. Hypoxic stress selectively reshapes corneal clock gene rhythms, with peak disruption coinciding with epithelial defects, reduced nerve density, and neutrophil infiltration (57). In the conjunctiva, SJL downregulates the BMAL1–REV-ERBα axis, thereby derepressing IL-17 transcription and promoting Th17-associated inflammation; melatonin restores this axis and suppresses IL-17 expression (47). Hypomethylation of PER2 and PER3 promoters in experimental dry eye models further indicates that inflammatory environments can epigenetically modify clock components (61).

Notably, clock disruption is not merely upstream of inflammation but engages in bidirectional interactions. IL-17 and the NF-κB pathway can directly inhibit BMAL1/CLOCK transcriptional activity, establishing a feedforward loop in which circadian misalignment promotes low-grade inflammation and metabolic stress, which in turn further suppress circadian regulation (56, 61).

### 4.2 Midstream: convergence of metabolic and immune signals and inflammatory amplification

Across models, oxidative stress emerges as a central hub linking circadian misalignment to inflammatory responses. Both mitochondrial dysfunction in the lacrimal gland and environmental stress in corneal and conjunctival epithelia (57) converge on ROS accumulation, which functions as both a marker of metabolic imbalance and a potent inflammatory trigger.

In lacrimal gland models combining sleep deprivation and silica nanoparticle exposure, a complete ROS–NLRP3–JAK2/STAT3–NF-κB–IL-17A signaling axis has been directly validated. Circadian disruption and particulate stress synergistically induce ROS accumulation, trigger NLRP3 inflammasome assembly, and activate JAK2/STAT3 and NF-κB p65 phosphorylation, resulting in marked upregulation of IL-17A. Activation of this axis is directly associated with lacrimal gland atrophy and reduced secretion (48). Although NLRP3 activation can promote gasdermin D–dependent pyroptosis, direct evidence for specific cell death modalities in SJL-related ocular models remains limited and should be interpreted cautiously (64).

Other inflammatory pathways may also participate. The TLR4/MyD88/NF-κB axis is activated in diabetic dry eye, and its inhibition attenuates corneal inflammation (49). In nonalcoholic steatohepatitis models, melatonin suppresses both TLR4/NF-κB signaling and NLRP3 activation (50), suggesting that circadian regulators integrate with innate immune pathways under metabolic stress. The cGAS–STING pathway, which senses cytosolic mitochondrial DNA, has been implicated in dry eye–associated inflammation (51, 52), and sleep deprivation–induced mtDNA release has been shown to activate cGAS–STING signaling in prostatitis models (53), providing a mechanistic analogue linking circadian disruption to innate immune activation.

Ferroptosis represents another potential, yet unverified link. Corneal tissues from dry eye models display lipid peroxidation, glutathione depletion, mitochondrial structural abnormalities, and differential expression of ferroptosis-related genes including ARNTL (Bmal1). In traumatic brain injury, BMAL1 downregulation enhances neuronal susceptibility to ferroptosis, whereas ferroptosis inhibition partially restores clock gene expression (55). Although direct evidence connecting SJL to ocular ferroptosis is lacking, these findings support a plausible hypothesis that clock disruption may increase epithelial vulnerability to ferroptotic stress.

### 4.3 Downstream: collapse of ocular surface homeostasis and a self-reinforcing loop

Sustained metabolic and inflammatory stress ultimately disrupts the three principal systems maintaining ocular surface integrity. In the lacrimal gland, combined clock dysregulation, oxidative injury, and inflammatory signaling produce acinar vacuolization and atrophy, with irreversible declines in basal and reflex tear secretion, accompanied by structural degeneration (39, 45, 46). At the molecular level, BMAL1 directly regulates transcription of ITPR2/3, linking clock disruption to impaired secretory capacity (56). In the meibomian gland, loss of rhythmic NAD^+^ synthesis suppresses the activity of the NAD^+^-dependent enzyme 3β-hydroxysteroid dehydrogenase, reducing local androgen production and promoting acinar atrophy, ductal hyperkeratinization, and altered lipid composition (58–60). Concurrent loss of corneal MUC4 expression weakens tear film anchoring (39). IL-17-driven disruption of epithelial tight junctions, together with an unstable lipid layer, accelerates evaporation, resulting in hyperosmolar stress and sensory nerve exposure (65).

These pathological changes extend beyond local tissue damage. Photophobia and chronic ocular discomfort lead to reduced daytime outdoor activity, increased nighttime screen exposure, and delayed sleep onset (66). Progressive deterioration in sleep quality further impairs central–peripheral circadian synchronization, reinforcing the underlying clock misalignment (67). Through this process, an initially reversible circadian disturbance becomes embedded within a self-sustaining cycle linking environmental stress, multitissue dysfunction, clinical symptoms, and maladaptive behaviors. The concept of “ocular social jetlag” introduced herein advocates for a fundamental paradigm shift in understanding DED. To crystallize the core dimensions of this shift, the traditional view is systematically contrasted with the emerging framework in **Table 2**.

**Table 2:** Conceptual shift in dry eye disease: from a structure–inflammation model to a circadian–immune–metabolic reprogramming framework.

Dimension	Traditional paradigm: structure–inflammation model	Proposed framework: circadian–immune–metabolic reprogramming	Conceptual implications and testable questions
Upstream drivers	Age-related gland atrophy, autoimmune involvement, local inflammatory triggers	Persistent circadian misalignment associated with modern lifestyles (SJL, nighttime light exposure)	Repositioning systemic rhythm disturbance as a potential upstream modifier; requires population-level quantification of SJL–ocular associations
Core pathogenic logic	Tear deficiency or excessive evaporation leading to hyperosmolar stress and secondary inflammation	Disruption of clock-regulated immune and metabolic coordination, with inflammation acting as a downstream amplifier	Mapping the temporal and cell-type-specific reprogramming events requires advanced spatial and single-cell omics approaches.
Primary pathological units	Structurally impaired lacrimal and meibomian glands	Functionally coupled ocular surface units with intrinsic circadian regulation (cornea, conjunctiva, meibomian gland)	Emphasis moves from static structural damage to regulation of functional timing
Typical clinical presentation	Predominantly older individuals with concordant symptoms and structural findings	Increasingly observed in younger individuals with marked symptoms but limited structural abnormalities	Broadens the recognized clinical spectrum; supports rhythm-informed phenotypic stratification
Diagnostic emphasis	Single time-point signs and imaging-based structural assessment	Temporal variation in symptoms, blink behavior, and tear film stability	Developing validated tools for continuous, rhythmic assessment is a key translational challenge
Therapeutic orientation	Tear supplementation, topical anti-inflammatory therapy, mechanical gland interventions	Interventions targeting circadian alignment (light exposure, sleep, feeding timing) and time-sensitive pharmacological strategies	Suggests a shift from exclusive downstream symptom control toward upstream rhythm modulation; requires prospective clinical testing
Research perspective	Local ocular surface disorder	Ocular surface as a functionally accessible interface reflecting systemic circadian–immune–metabolic interactions	Ocular rhythmic signatures may offer novel, non-invasive biomarkers for broader metabolic and sleep-related disorders.

## 5 Digital immune phenotype: decoding the dynamic landscape of ocular surface circadian disruptions

The ocular surface integrates photoreception, immune defense, and optical transparency, positioning it as a potential noninvasive biosensor for the systemic clock-immune-metabolism network, which governs time-dependent immune and metabolic functions. The digital immune phenotype concept represents immune-related traits derived from rhythmic data, translating social jetlag effects into a quantifiable dynamic map of ocular surface temporal dysfunctions. Although innovative, this concept remains largely conceptual, relying on emerging technologies, and requires clinical validation to substantiate the transition from signal detection to immune phenotyping.

### 5.1 From symptoms to mechanisms: continuous monitoring reveals desynchronization trajectories

Traditional diagnostics rely on subjective recall and single-time-point assessments, often missing dynamic disease progression. Key pathological events, such as diurnal reductions in meibomian gland lipid secretion and nocturnal accumulation of tear inflammatory mediators, vary continuously over time. Emerging technologies offer potential to capture rhythm parameters, including phase, amplitude, and waveform, but most remain experimental with limited validation in clinical dry eye populations.

For example, computer vision-based blink analysis focuses on the rhythmic entropy of blink intervals, a measure of interval regularity. Healthy individuals display longer but regular intervals (low entropy) during daytime visual tasks, whereas early dry eye patients show increasing irregularity (high entropy) with prolonged task duration, reflecting impaired rhythmic buffering in neural feedback loops for surface hydration and consequent decompensation (68, 69). These preliminary findings provide a foundation for dynamic monitoring but require larger clinical studies for confirmation. Wearable tear sensors, such as fluorescent hydrogel patches, aim to map 24-hour profiles of immune molecules like lysozyme and MMP-9. A recent fluorescent MOF hydrogel patch achieved high-sensitivity lysozyme detection (limit 1.5 nM) analyzable via smartphone apps (70), though long-term validation in dry eye patients is pending. Multichannel nanophotonic immunosensors, nanowire field-effect transistors, and antibody microarrays enable ultrasensitive, real-time multiplex detection (e.g., MMP-9, lysozyme, lactoferrin) with minimal sample requirements and ease of use (71–73). Despite these promising proofs-of-concept, a significant gap remains between laboratory-scale engineering performance and robust clinical evidence. Current wearable tear sensors are primarily validated in small cohorts or healthy subjects over short durations, failing to capture the long-term heterogeneity of DED populations (71). Technologically, the translation from prototype to clinic faces several critical barriers. First, the limited tear volume and its high susceptibility to evaporation and environmental noise make precise quantification difficult; furthermore, the very presence of a sensor may trigger reflexive tearing, diluting biomarker concentrations and introducing sampling bias (70). Second, sensor-tissue interfaces are prone to bio-fouling, the non-specific adsorption of proteins and lipids, which leads to signal drift and reduced stability during 24-hour monitoring (74). Third, achieving reliable multiplexing without signal crosstalk remains an engineering challenge for simultaneous detection of cytokines like MMP-9 and lysozyme. Beyond technical hurdles, the path to clinical adoption requires scaling from nanofabrication to GMP-compliant mass production and navigating complex regulatory frameworks such as FDA or ISO certifications (75). Consequently, the direct linkage between these digital signals and specific immune phenotypes remains conceptual and awaits rigorous validation in multi-center clinical trials.

### 5.2 Multimodal data fusion: constructing personalized circadian disruption networks

Circadian disruptions represent cross-system, multi-timescale desynchronization processes. Constructing a digital immune phenotype therefore emphasizes reconstructing synchronization across multimodal time series rather than isolated metrics. Due to current technological limitations, this relies on accessible neurophysiological, behavioral, and systemic rhythm signals, not direct continuous measurements of local ocular surface rhythms, making the signal-to-phenotype translation a conceptual framework.

Video-based facial and ocular analysis reliably extracts blink frequency, eyelid closure dynamics, and heart rate, aligning with contact sensors and distinguishing resting from task-loaded states (76, 77). These metrics reflect the dynamic regulatory state of the central-autonomic nervous system and serve as a feasible, remote, non-invasive entry point for rhythm monitoring, but they are not direct proxies for the tear film or ocular surface immune microenvironment.

In chronomedicine, wearable devices collect longitudinal activity-rest rhythms, heart rate variability, sleep, and light exposure data, quantified using parametric and nonparametric methods to assess phase, amplitude, and stability (78–80). These approaches are primarily derived from systemic chronomedicine and inflammatory disease studies, with limited direct ocular evidence; thus, they serve as referential analogies rather than confirmations. Integrating data across temporal resolutions and biological levels is critical to understanding circadian disruptions (79). Accordingly, ocular signals function as observable nodes in systemic rhythm networks, not independent local clocks. Coordinated changes in pupillary dynamics, oculomotor parameters, and heart rate variability reveal autonomic response patterns and stress adaptation subtypes, suggesting that circadian disruptions manifest as multimodal signal coupling reorganizations beyond single-metric anomalies (81).

In summary, current personalized circadian disruption networks integrate ocular neurobehavioral signals with systemic rhythm parameters over time. Analyses of synchrony and phase relationships aim to explore social jetlag-associated rhythm reprogramming. Digital mapping of tear film and ocular surface immune rhythms awaits advances in sensing technologies and physiological validation to strengthen the rationale for linking signal detection to immune phenotyping.

## 6 Chronotherapy and future interventions: targeting the immune clock in dry eye management

Viewing circadian disruption as an upstream driver of ocular surface immune disequilibrium provides a new entry point for the management of DED. Future strategies may shift from purely symptomatic local treatment toward multidimensional interventions aimed at restoring immune circadian synchrony. According to the maturity of current evidence, these strategies can be categorized into optimization of drug timing based on symptom and immune fluctuations, direct targeting of clock immune hub molecules, modulation of systemic immunity through environmental zeitgebers, and integrative chronomedicine-based management.

### 6.1 Targeting the nocturnal window: symptom-based optimization of drug timing

DED symptoms and ocular surface inflammatory markers exhibit diurnal variation (82, 83). Aligning anti-inflammatory treatment with these inflammatory windows may therefore enhance efficacy while reducing adverse effects. Drawing on established chronotherapeutic approaches in systemic immune inflammatory diseases such as rheumatoid arthritis (84), we propose the hypothesis that administration of intermediate or long-acting topical formulations, for example gels or liposomes, at bedtime, targeting nocturnal innate immune activation in DED, may more effectively suppress the morning inflammatory peak while reducing cumulative daytime exposure. Although direct evidence in DED is currently lacking, a recent study reporting the efficacy and safety of pulsed low dose hydrocortisone in Sjögren’s syndrome related DED (85), provides preliminary support for the feasibility of this approach.

At present, melatonin is the circadian-related molecule with the greatest translational potential. In addition to pineal secretion, melatonin is locally synthesized at the ocular surface and signals through MT1 and MT2 receptors, forming a relatively independent regulatory system (86). Multiple preclinical studies have demonstrated that melatonin attenuates inflammation, oxidative stress, and glandular dysfunction in DED models (**Table 3**). A pilot clinical study in primary Sjögren’s syndrome associated DED showed that oral melatonin at 5 mg per day for 8 weeks improved tear secretion, tear film stability, and symptom scores, while reducing serum IL-6 levels with good tolerability (93), providing human evidence for its systemic immunomodulatory potential. In parallel, delivery platforms such as nanoliposomes and drug eluting contact lenses are being explored to enhance ocular bioavailability and retention.

**Table 3:** Immunomodulatory effects of melatonin in dry eye disease and supporting evidence.

Study	Model/subjects	Key immunological findings	Proposed mechanisms/contributions	Major Limitations
Cai Y (87)	High-fat diet mice	Shift macrophages M1→M2, ↓ERK/JNK	Upregulation of IFT27; MAPK signaling inhibition	Preclinical mouse model; IFT27 causal role associative
Wang C (88)	Aged mouse model	Suppressed NLRP3 inflammasome activation in the lacrimal gland and reduced pyroptosis	Activation of the SIRT1 signaling pathway	Preclinical animal model
Liu R (89)	Human meibomian gland epithelial cells (*in vitro*)	Reduced expression of pro-inflammatory cytokines and enhanced lipid synthesis	Inhibition of MAPK/NF-κB signaling pathways	*In vitro* study
Wang B (90)	Cell culture and mouse models	Attenuated oxidative stress, restored autophagy, and protected ocular surface epithelium	Induction of HO-1 expression	Animal model; non-topical administration
Lou Q (91)	BAC-induced rat dry eye model	TAT-modified liposomal melatonin markedly alleviated corneal inflammation and epithelial pyroptosis	Enhanced corneal penetration and suppression of NLRP3/GSDMD signaling	Animal model; preservative-induced injury
Navarro-Gil (92)	Rabbit tear secretion model	Melatonin-loaded contact lenses produced greater tear secretion than eye drops	Significantly prolonged ocular surface retention time	Animal model
Mandić B (93)	pSS-associated DED patients (n = 12)	Oral melatonin improved clinical signs and symptoms and significantly reduced serum IL-6 levels	Provides preliminary clinical evidence for systemic immunomodulatory effects	Small sample size; requires validation in randomized controlled trials

By contrast, direct targeting of core clock transcription factors such as REV-ERB and RORα remains at an early stage. Animal studies suggest that REV-ERB agonists can alleviate LPS-induced ocular inflammation (94), while inverse agonists of RORα or γt improve glandular function in Sjögren’s syndrome models (95). Moreover, downregulation of lacrimal gland Nr1d2 in postoperative DED mice leads to lipid metabolic disturbances, which can be partially reversed by the agonist SR9011 (46). These findings indicate that core clock pathways may represent upstream therapeutic targets in specific DED contexts, although their translational value requires systematic validation.

### 6.2 Modulating environmental zeitgebers: hypothesis-generating lifestyle interventions

Management of the two strongest zeitgebers, light exposure and feeding time, aims to stabilize the overall biological clock and may indirectly attenuate systemic immune inflammation. Nocturnal artificial light suppresses melatonin secretion, disrupts clock gene expression, and is associated with low grade systemic inflammation (96). Therefore, for DED patients with social jetlag, increased daytime exposure to natural light and reduction of blue light exposure before bedtime are suggested (97–99). Although there is no direct clinical evidence in DED, such measures may create a more favorable internal environment for ocular surface repair by improving sleep and global immune balance.

In addition, time-restricted eating effectively synchronizes peripheral metabolic clocks and improves metabolic inflammation (100). In DED patients with metabolic risk factors such as MGD, time restricted eating may indirectly support meibomian gland function by optimizing systemic lipid metabolism and inflammatory status (101). At present, these interventions remain hypothesis generating strategies due to the lack of direct clinical evidence in DED.

Future management paradigms may integrate digital health technologies. Continuous passive collection of behavioral data including sleep, activity, and light exposure via wearables and smartphones, combined with patient reported outcomes and even home based ocular surface monitoring, could enable construction of individualized circadian health profiles (102). On this basis, artificial intelligence algorithms may identify patterns of circadian misalignment and deliver real time, context specific micro interventions, for example stand up and seek natural light or reduce screen exposure 30 minutes earlier tonight, forming a quantifiable, feedback driven monitor intervene optimize loop (103). This approach may overcome the limitations of conventional lifestyle advice and provide a sustainable strategy for chronic DED management.

### 6.3 Discussion and limitations: considerations of clinical heterogeneity

It must be acknowledged that the circadian centered framework presented here is primarily supported by evidence derived from relatively young patient populations characterized by lifestyle related immunometabolic dysregulation, such as excessive digital screen use, sleep deprivation, and social jetlag. These patients typically present with prominent symptoms but relatively mild structural abnormalities, for example limited meibomian gland atrophy or lacrimal gland damage. In this circadian sensitive DED subtype, chronobiological interventions may be most effective.

However, DED is highly heterogeneous. In classical subtypes driven predominantly by overt structural damage, such as lacrimal gland fibrosis in advanced Sjögren’s syndrome or severe cicatricial meibomian gland dysfunction, circadian disruption is more likely to act as a concomitant or aggravating factor rather than a primary driver. In these settings, circadian interventions should be positioned as adjunctive and supportive strategies aimed at optimizing overall health, improving sleep quality, and potentially enhancing responsiveness to baseline therapies, such as immunosuppressants or physical treatments, rather than replacing conventional approaches.

Looking ahead, several key evidence gaps must be addressed. First, longitudinal studies across different DED etiologies and severities are needed to validate core clock gene expression patterns and to delineate regulatory networks within specific ocular surface immune cell populations. Second, rigorously designed randomized controlled trials targeting melatonin and other clock modulating molecules are required to establish efficacy and safety. Finally, the development and validation of user friendly, clinically applicable tools for circadian assessment and management are essential for translating this emerging concept into routine practice. Only through such stepwise investigation can chronobiology make a substantive contribution to precision and personalized therapy in DED.

## Data Availability

The raw data supporting the conclusions of this article will be made available by the authors, without undue reservation.
